# Medicinal plants – prophylactic and therapeutic options for gastrointestinal and respiratory diseases in calves and piglets? A systematic review

**DOI:** 10.1186/s12917-016-0714-8

**Published:** 2016-06-06

**Authors:** Hannah Ayrle, Meike Mevissen, Martin Kaske, Heiko Nathues, Niels Gruetzner, Matthias Melzig, Michael Walkenhorst

**Affiliations:** Department of Livestock Sciences, Research Institute of Organic Agriculture (FiBL), Ackerstrasse 113, postbox 219, Frick, 5070 Switzerland; Division Veterinary Pharmacology & Toxicology, Department Clinical Research and Veterinary Public Health, Vetsuisse Faculty, University of Bern, Laenggassstrasse 124, Bern, 3012 Switzerland; Department of Farm Animals, Vetsuisse Faculty, University of Zurich, Winterthurerstrasse 260, Zurich, 8057 Switzerland; Department of Clinical Veterinary Medicine, Swine Clinic, Vetsuisse Faculty, University of Bern, Bremgartenstrasse 109a, Bern, 3012 Switzerland; Dahlem Centre of Plant Sciences, Institute of Pharmacy, Freie Universität Berlin, Koenigin-Luise-Strasse 2 + 4, Berlin, 14195 Germany

**Keywords:** Medicinal plants, Calves, Piglets, Gastrointestinal diseases, Respiratory diseases

## Abstract

**Background:**

Gastrointestinal and respiratory diseases in calves and piglets lead to significant economic losses in livestock husbandry. A high morbidity has been reported for diarrhea (calves ≤ 35 %; piglets ≤ 50 %) and for respiratory diseases (calves ≤ 80 %; piglets ≤ 40 %). Despite a highly diverse etiology and pathophysiology of these diseases, treatment with antimicrobials is often the first-line therapy. Multi-antimicrobial resistance in pathogens results in international accordance to strengthen the research in novel treatment options. Medicinal plants bear a potential as alternative or additional treatment. Based on the versatile effects of their plant specific multi-component-compositions, medicinal plants can potentially act as ‘multi-target drugs’. Regarding the plurality of medicinal plants, the aim of this systematic review was to identify potential medicinal plant species for prevention and treatment of gastrointestinal and respiratory diseases and for modulation of the immune system and inflammation in calves and piglets.

**Results:**

Based on nine initial sources including standard textbooks and European ethnoveterinary studies, a total of 223 medicinal plant species related to the treatment of gastrointestinal and respiratory diseases was identified. A defined search strategy was established using the PRISMA statement to evaluate 30 medicinal plant species starting from 20’000 peer-reviewed articles published in the last 20 years (1994–2014). This strategy led to 418 references (257 *in vitro*, 84 *in vivo* and 77 clinical trials, thereof 48 clinical trials in veterinary medicine) to evaluate effects of medicinal plants and their efficacy in detail. The findings indicate that the most promising candidates for gastrointestinal diseases are *Allium sativum* L., *Mentha x piperita* L. and *Salvia officinalis* L.; for diseases of the respiratory tract *Echinacea purpurea* (L.) MOENCH, *Thymus vulgaris* L. and *Althea officinalis* L. were found most promising, and *Echinacea purpurea* (L.) MOENCH, *Camellia sinensis* (L.) KUNTZE, *Glycyrrhiza glabra* L. and *Origanum vulgare* L. were identified as best candidates for modulation of the immune system and inflammation.

**Conclusions:**

Several medicinal plants bear a potential for novel treatment strategies for young livestock. There is a need for further research focused on gastrointestinal and respiratory diseases in calves and piglets, and the findings of this review provide a basis on plant selection for future studies.

**Electronic supplementary material:**

The online version of this article (doi:10.1186/s12917-016-0714-8) contains supplementary material, which is available to authorized users.

## Background

A high standard of animal health and welfare is striven in modern livestock husbandries. Health in early life represents a precondition for a superior constitution and results in a high productivity later in life. The mammalian immune system is still immature in the first weeks of life and, in combination with an inappropriate colostral supply, contact to pathogens often results in high morbidity and mortality in young farm animals. Inadequate management including long distance transports, fasting, commingling of individuals from different sources, abrupt changes in diet or incorrect diet, overcrowding of pens, improper climate and suboptimal hygiene are crucially involved in infectious diseases [1–6]. In calves and piglets, the first contact sites for pathogens are the epithelia of the gastrointestinal and respiratory tract. Table 1 shows four of the most important infectious disease complexes in calves and piglets leading to a decreased animal performance and welfare and therefore high economic losses.

**Table 1:** Challenging infectious diseases in calves and piglets: pathogens, pathophysiology, resulting demands for prophylaxis and therapy

Disease complex	Pathogens			Pathophysiology/pathogenesis	Demands for prophylaxis and therapy
	Bacteria	Viruses	Parasites		
Calves					
Neonatal Calf Diarrhea^1^	*Escherichia coli*	Bovine Coronavirus	*Cryptosporidium parvum*	secretory/malabsorptive/maldigestive diarrhea	antimicrobial
		Rotavirus		dehydration	antiviral
				hypovolemic shock	antidiarrheal
				decrease in temperature	antiadhesive
				d-lactat acidosis	astringent
				septicaemia	spasmolytic
				neurological symptoms	analgesic
				apathy	anti-inflammatory
				recumbency	orexigenic
				reluctance to drink	prebiotic
				fever	immunostimulant
Bovine Respiratory Disease^2^	*Mannheimia haemolytica*	Infectious bovine rhinotracheitis virus		catharral/interstitial/fibrinous bronchopneumonia	antimicrobial
	*Pasteurella multocida*	Parainfluenza type 3 virus		fever	antiviral
	*Histophilus somni*	Bovine respiratory syncytial virus		increased respiratory rate	analgesic
	*Mycoplasma bovis*	Bovine viral diarrhea virus		dyspnoea	anti-inflammatory
				inappetence	immunostimulant
				nasal discharge	mucolytic
				coughing	secretolytic
				apathy	antitussive
				runting	
Piglets					
Neonatal Diarrhea	*Escherichia coli*	Rotavirus	*Cryptosporidium spp.*	secretory/malabsorbtive/maldigestive diarrhea	antimicrobial
Postweaning Diarrhea^3^	*Clostridium perfringens*	Coronavirus	*Isospora suis*	enteritis	antiviral
	*Lawsonia intracellularis*	Porcine Circovirus type 2	*Trichuris suis*	colitis	antidiarrheal
	*Brachyspira spp.*		*Oesophagostomum dentatum*	dehydration	antiadhesive
	*Salmonella spp. *			acidosis	astringent
	*(Yersinia spp.)*			septicaemia	spasmolytic
				neurological symptoms	analgesic
				apathy	anti-inflammatory
				reduced growth rates	orexigenic
					prebiotic
					immunostimulant
Porcine respiratory disease complex^4^	*Pasteurella multocida*	Porcine reproductive and respiratory syndrome virus		suppurative/fibrinous/interstitial bronchopneumonia	antimicrobial
	*Mycoplasma hyopneumoniae*	Swine influenza virus		coughing	antiviral
	*Actinobacillus pleuropneumoniae*	Porcine circovirus type 2		nasal discharge	analgesic
	*(Streptococcus suis)*			increased respiratory rate	anti-inflammatory
	*(Haemophilus parasuis)*			fever	immunostimulant
				reduced growth rates	mucolytic
				runting	secretolytic
					antitussive

^1^[10–13] ^2^[3–5, 23, 25] ^3^[6, 15–18] ^4^[22, 27, 29]

In calves and piglets, a variety of pathogens can cause gastrointestinal diseases. Neonatal calf diarrhea represents the most frequent cause of calf losses [2, 7–9] with a mortality of around 55 % in the U.S.A. and in Korea [10] and a morbidity ranging from 12 % in the U.S.A., 23 % in Canada up to 53 % in The Netherlands [1, 7, 11]. Insufficient colostral supply and failure of feeding or improper diet are triggers for diarrhea in calves [12–14]. In suckling and postweaning piglets an infection with enterotoxigenic *Escherichia coli* strains has been reported to lead to high economic losses as a result of a constant high morbidity and mortality [15]. Verocytotoxin-producing *Escherichia coli* infections can lead to more seldom but severe edema disease in weaned pigs [16–18]. The prevalence of postweaning diarrhea has been reported to be 35 % in France [19], the morbidity was stated to exceed 50 % in Finland [20] and the mortality can be as high as 25 % without therapy [17]. The incidence of neonatal diarrhea in piglets depends on concentration of antibodies in sow’s colostrum. While piglets are protected by the antibodies in sow’s milk, the predisposition for postweaning diarrhea increases with weaning. Additional factors to the immunological gap, including abrupt changes in diet, an increase in stomach pH, and changes in the enzymatic and cellular configuration of the intestine lead to dysbiosis [6, 17, 21].

Respiratory diseases in calves and piglets have been assessed as one of the most serious diseases with regard to financial losses because of decreased weight gain, costs for veterinary interventions and higher condemnation at slaughter [22]. In fattening calves bovine respiratory disease is a considerable challenge with a morbidity ranging from 49 % in Switzerland to 80 % in the U.S.A. [23, 24]. There is a disposition of the bovine respiratory tract to respiratory diseases. Improper microclimate, noxious gases and distress through transportation are predisposing factors additionally [25, 26]. Respiratory diseases are also of high importance in pigs. The morbidity rates differ between countries; a morbidity of 10 and 40 % have been reported for Denmark and the U.S.A. respectively [27, 28]. Mortality rates up to 15 % [29] have been reported and attributed with the porcine respiratory disease complex. The interaction of various pathogens as well as housing conditions, management and genetic factors, were reported to cause bronchopneumonia [27, 29].

Antibiotic therapy is often the first-line therapy of diseases of the gastrointestinal and respiratory tract in calves and piglets. A previously published study showed that fattening calves receive antibiotics for 30 days on average. Moreover, calves are frequently treated with reserve antibiotics such as fluorchinolones and cephalosporines of the 3. and 4. generation [30]. In pig production, routine pro- and metaphylactic administration of antimicrobial agents is a widely-used practice in herds suffering from neonatal diarrhea, postweaning diarrhea or edema disease irrespective of increasing ineffectiveness in consequence of bacterial resistance [6]. More than 60 % of the antibiotics used in porcine husbandry are administered by oral group treatment [31]. Data on antimicrobial resistance monitoring indicated that 59 % of porcine *Escherichia coli* strains from fecal samples showed resistance to at least one antibiotic and 12 % to more than four of the antibiotics that were investigated [32]. With regard to increasing antimicrobial resistance worldwide, the prevailing issue of reducing antibiotics in food producing animals is seeking for novel options to prevent and cure most common and costly diseases. Improved biosecurity and housing conditions, new feeding regimes, vaccinations and the use of disease-resistant breeds are important provisions.

The diverse etiopathology and symptomatology of diseases in young stock is a challenge and demands a multi-targeted therapy. In contrast to chemically synthesized mono-target drugs, multi-target drug characteristics are typical for medicinal plants based on their multi-component composition, which can lead to pleiotropic, synergistic or additive effects in the organism [33, 34]. The broad spectrum of natural products from plants represents a prevailing and widely unemployed potential especially for medication of herbivore and omnivore livestock [35]. Medicinal plants have been used worldwide for prevention and treatment of diseases in human and animals for centuries. Ethnoveterinary research and the underlying documents describing traditional and recent use of medicinal plants [36–38] could be exploited as alternative or as supportive tools to reduce the use of antibiotics in livestock. Additionally, some medicinal plants are known to modulate the immune system and inflammation and could be used as a prophylaxis for infectious diseases.

Human clinical studies, experimental *in vivo*, *ex vivo* and *in vitro* studies on medicinal plants are available, but there is a lack of comprehensive research for veterinary medicine, especially in young farm animals. Therefore, the purpose of the underlying work is to gain information about potential efficacy of medicinal plants in human and veterinary medicine including *in vitro*, *in vivo* and clinical studies.

The aim of this systematic literature review is to identify medicinal plant species or their extracts that are promising candidates for use in diseases of the gastrointestinal and respiratory tract and for stimulation of the immune system and prevention or therapy of inflammation in calves and piglets. Candidate plants should bear a reliable potential for effective treatment and prevention of these diseases. The information obtained can build a basis for state-of-the-art experimental trials and clinical studies with medicinal plants of interest for the treatment of gastrointestinal and respiratory diseases and for the modulation of the immune system and inflammatory processes in calves and piglets.

## Methods

The design of the systematic review was ‘a priori’ individually developed according to the recommendations of the PRISMA statement [39, 40] and AMSTAR measurement tool [41]. The research question was designed following the PICOS scheme [39]: the *population* are calves and piglets in livestock farming, the *intervention* is a treatment with medicinal plants, the *comparator* is no treatment, a placebo or standard treatment, the *outcome* is the effect of the plant, the *study design* included *in vitro*, *ex vivo*, *in vivo* and clinical trials. The detailed protocol of the systematic review is provided in the Additional file 1.

### Selection of plant species

To choose eligible plant species, different *initial sources* were screened in respect to plant species frequently recommended for the treatment of gastrointestinal diseases, particularly unspecified or infectious diarrhea and gastrointestinal spasms (QA) and respiratory diseases (QR) as well as those plants that have been reported to modulate the immune system and affect inflammation in infectious diseases (QL). Regarding the intended use of medicinal plants in Western livestock husbandry, potential plant species should be economically available or easy to cultivate in Europe. The *initial sources* included standard literature, based on traditional empiric knowledge and historical literature of veterinary [42–45], and human phytotherapy [46], peer-reviewed publications of European [47] and in particular Swiss ethnoveterinary medicine [36, 38] and a report of the European Food Safety Authority (EFSA) [48] focusing on the use of plants as feed additives in animal production. All plant species of these sources connected to one or more of the indications were recorded including the used part(s) of plant, the route of administration, the dosage, the contraindications and adverse effects (Additional file 1). Based on the plant species that had been mentioned in at least three different *initial sources* for the same indication, a preliminary selection was established. This selection was sent to three specialists in European veterinary phytotherapy to capitalize their experience. The experts were asked to comment on the preliminary selection of plant species regarding the most common ones being particularly suitable for treatment and prevention of gastrointestinal and respiratory diseases.

### Selection of scientific references

#### Bibliographic search

The chosen plant species were included in the following step. A bibliographic web-based search was conducted based on the recommendations of the PRISMA statement [39, 40] and AMSTAR measurement tool [41]. An introduction in scientific bibliographic literature searches and continuous support was provided by a professional librarian. The bibliographic sources used included PubMed [49] and Web of Science [50]. Both were consulted in the time between 2015-02-16 and 2015-02-19 by one person. The search terms consisted of the Latin name, the common trivial name in English and the pharmaceutical denomination in Latin (e.g. “Foeniculum vulgare” OR “fennel” OR “foeniculi fructus”). In the PubMed keyword search, the results were refined with the *subjects* ‘complementary medicine’, ‘dietary supplements’, ‘systematic review’, ‘toxicology’ and ‘veterinary science’. In the PubMed search with MeshTerms, only the Latin name of the plant was used and the *subheadings* ‘adverse effects’, ‘analysis’, ‘drug effects’, ‘microbiology’, ‘pharmacology’, ‘therapeutic use’, ‘therapy’ and ‘toxicity’. In the Web of Science database, the search was conducted in the *research domain* ‘science technology’ and the results were refined with the *research areas* ‘pharmacology’, ‘infectious diseases’, ‘toxicology’, ‘veterinary sciences’, ‘microbiology’, ‘gastroenterology’, ‘integrative complementary medicine’, ‘general internal medicine’, ‘respiratory system’ and ‘virology’. Overall, only peer-reviewed articles written in English or German language and published between 1994 and 2014 were considered for further evaluation to ensure contemporary scientific quality and timeliness of the review. The references found were saved in an EndNote X7 data base [51] and the information on each plant was stored in a separate folder in this database. Duplicates were removed for each plant species.

In some studies more than one plant species was investigated (e. g. a screening of plant species against *E. coli* [52]). In other studies, more than one indication was considered and investigated (e. g. spasmolytic effect of *Plantago lanceolata* L. on intestine and trachea [53]). Therefore, the following definition of “reference” was introduced:

reference = indication per plant species per peer-reviewed publication

#### Term-list search

A term-list search was conducted within each plant species using the search function in EndNote X7 [51]. Only references containing one of the following predefined keywords occurring in the title or the abstract were included: ‘pig*’, ‘calv*’, ‘muco*’, ‘spasmo*’, ‘anti*’ (e.g. antimicrobial, antibacterial, antiviral, antifungal, antioxidant, antinociceptive…), ‘wean*’, ‘intest*’, ‘gastro*’, ‘pulmo*’, ‘broncho*’, ‘pharma*’, ‘eff*’(e.g. efficiency, effectivity, effect), ‘bioactiv*’, ‘constitu*’. References containing the terms ‘tumor’ or ‘cancer’ were excluded. The check of the excluded references lowered the risk to exclude relevant references.

#### Refining with inclusion and exclusion criteria

The remaining references were refined using a selective screening of the title. References remained if the content suffices the objective of the review. Therefore, inclusion criteria were pre-defined by two scientists and lead to an inclusion of all references containing investigations of plants in *in vitro*, *ex vivo*, *in vivo* or clinical studies. Besides these categories, the evaluation included the following inclusion criteria: antibacterial effect, enhancement of antibiotics, antiviral effect, antiprotozoal effect, anti-inflammatory effect, analgesic effect, spasmolytic effect, antiadhesive effect, astringent effect, secretolytic or mucolytic effect, antitussive effect, and other effects on the gastrointestinal tract, respiratory tract or immune system, treatment of diarrhea, bacterial or viral infections of the gastrointestinal tract or respiratory tract, bronchopneumonia, common cold, cough as well as ingredients, constituents, components of plants and the detection or extraction of them, toxic activity or adverse effects due to a treatment with plants.

Exclusion criteria were chosen in order to exclude references dealing with other plant species or subspecies than those we focused on, a mixture of different plant species investigated as one single preparation, pathogens affecting only humans, diseases regulated by laws, cultivation or breeding of plants, plant genetics, seeds and fertilizers, regional reservoirs, habitats or demands for growing of plants, plant pathology, plant protection systems or pesticides, ecology, geology, ethology, sociology, the usage of the plant as food, food technology or food-packaging, the use of the plant as a repellent or insecticide, other medical branches, other diseases or apparatuses than mentioned in the inclusion criteria (e.g. dermatology, cardiology, oncology, nephrology, diabetes) as well as other animal classes than mammalians and birds.

#### Classification

Thereby the references were classified into different categories of trial types. Studies investigating diseases occurring naturally in the investigated animal species or in humans were categorized as ‘clinical references’. Trials investigating diseases or the effect of plants in animal models were categorized as ‘*in vivo* references’. Studies using pathogens, cell layers or *ex vivo* models were categorized as ‘*in vitro* references’. Studies investigating the pharmacologic characteristics, constituents or the detection of them were categorized as ‘pharmacognostic references’ and the evaluation of plants summarizing other studies as ‘review references’.

In the last step, abstracts of the remaining clinical, *in vivo* and *in vitro* references were studied by one person. During this process, further references were excluded because they did not match the predefined selection criteria.

#### Assessment of clinical, *in vivo* and *in vitro* references

The remaining references were assessed by the following characteristics: used plant species, type of reference (clinical, *in vivo* or *in vitro*), indication of the reference inspired by the ATCvet classification (QA, QR, QL) [54], animal species used, study design, pharmaceutical form of the plant, type of application, concentration tested, dosage or minimal inhibition concentration and, if available, the tested pathogen.

To assess the potential of the selected plant species, a reconciliation of the demands for prophylaxis and therapy of gastrointestinal and respiratory diseases with the hypothesized and tested effects of the plants was performed. The demands for prophylaxis and therapy were derived from the pathophysiology of the focused diseases (Table 1). According to these data, plant-derived treatment options should act bacteriostatic or bactericidal, synergistically with antibiotics, antiviral, antiprotozoal, anti-inflammatory, analgesic, immunomodulatory, antidiarrheal, antiadhesive, spasmolytic, astringent, expectorant or antitussive (depending on the indication). The conclusion of a trial on the investigated hypothesized effect of the plant species (Additional file 3) was transferred in the following assessment. To compare the potential of the plant species, a scoring system was established. For each significantly proven effect, the plant species one point was given, while for each uncertain effect, zero points were assigned, and for each disproved effect a point was subtracted (for more details see Additional file 1). Clinical studies were given more weight compared to *in vivo* studies followed by *in vitro* studies. Clinical studies were given a weight of three, *in vivo* studies two, and *in vitro* studies one. The weighted average of the sum of points of the clinical, *in vivo*, and *in vitro* scores served as the final score. The scores were used to identify the plant species that are the most efficacious options for related disease complexes.

$$ \begin{array}{l}\mathrm{Score} = 3\ \mathrm{x}\ \Big(\mathrm{number}\ \mathrm{of}\ \mathrm{proven}\ \mathrm{effects}\ \mathrm{in}\ \mathrm{clinical}\ \mathrm{studies}\ \hbox{--}\ \mathrm{number}\ \mathrm{disproof}\ \mathrm{of}\ \mathrm{effects}\\ {}\ \mathrm{in}\ \mathrm{clinical}\ \mathrm{studies}\left) + 2\ \mathrm{x}\ \right(\mathrm{number}\ \mathrm{of}\ \mathrm{proven}\ \mathrm{effects}\ \mathrm{in}\  in\  vivo\ \mathrm{studies}\ \hbox{-}\ \mathrm{number}\ \mathrm{of}\\ {}\mathrm{disproof}\ \mathrm{of}\ \mathrm{effects}\ \mathrm{in}\  in\  vivo\ \mathrm{studies}\left) + 1\ \mathrm{x}\ \right(\mathrm{number}\ \mathrm{of}\ \mathrm{proven}\ \mathrm{effects}\ \mathrm{in}\  in\  vitro\ \mathrm{studies}\\ {}\hbox{-}\ \mathrm{number}\ \mathrm{of}\ \mathrm{disproof}\ \mathrm{of}\ \mathrm{effects}\ \mathrm{of}\  in\  vitro\ \mathrm{studies}\Big)\end{array} $$

## Results

**Fig. 1:**
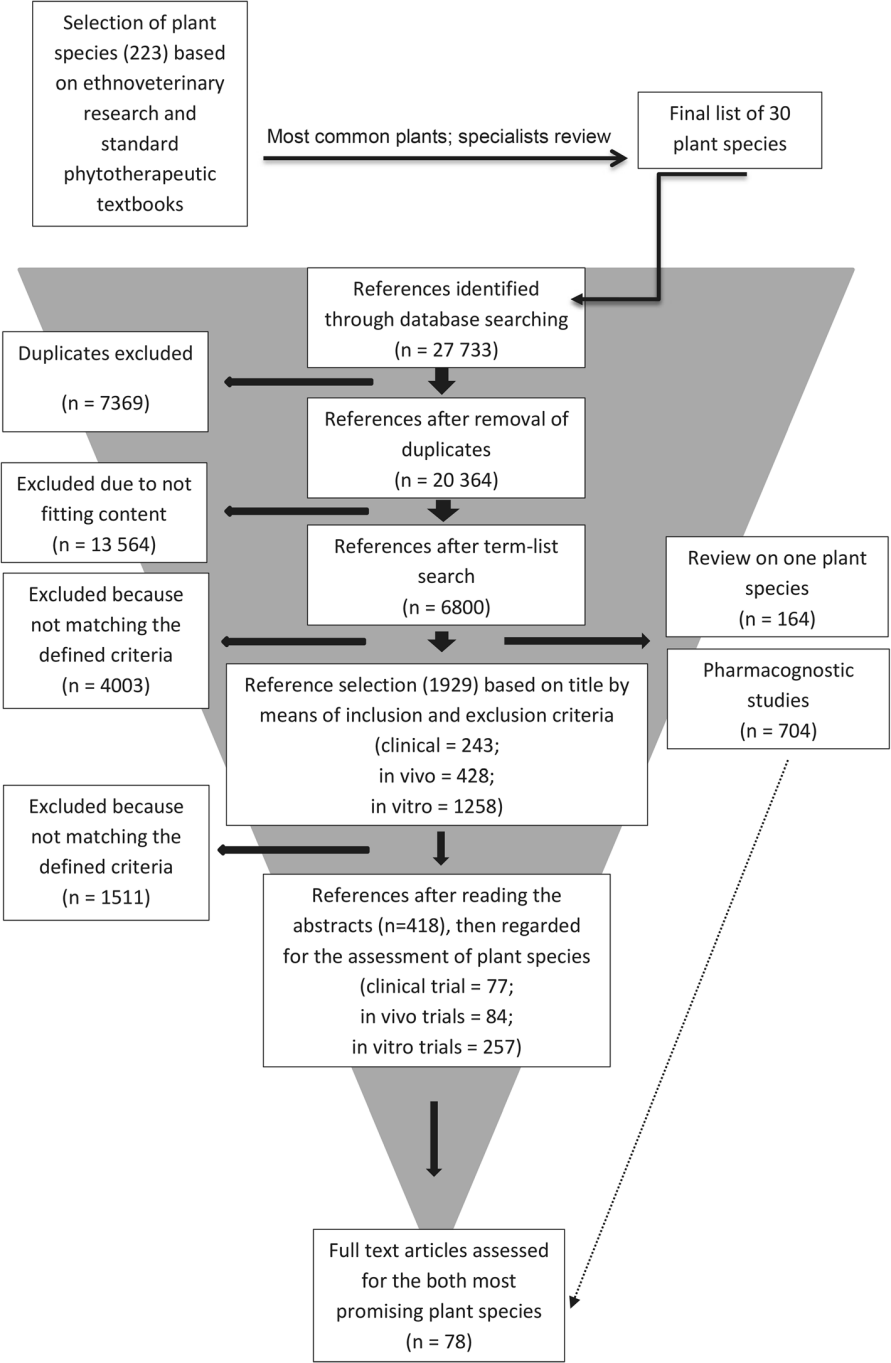
Process of the systematic literature review

The procedure of this systematic literature review is visualized in Fig. 1. The screening of ethnoveterinary research and standard phytotherapeutic textbooks (*initial sources*) led to a total of 223 plant species recommended for the treatment and prophylaxis of gastrointestinal (diarrhea and intestinal spasms) and respiratory diseases in human and animals. A number of 134 different plant species were recommended for QA, 121 for QR and 44 for QL (Additional file 2). A preliminary selection of 29 plant species, recommended in at least three different sources for the same indication, was established. Therefrom, 17 plant species were recommended for QA, 15 for QR and 8 for QL. The specialists review led to an addition of one plant species (*Origanum vulgare* L.) to the preliminary list including finally 30 plant species. All of these plant species meet the claims for cost-efficiency or cultivability in Europe.

In the subsequent bibliographic search 20,364 references (after removal of duplicates) were found for the 30 plants species (Table 2). During the term-list search, the amount of relevant references led to a reduction of references with 6,800 remaining references. An ensuing random check of the excluded references confirmed the selected terms. The subsequent screening of titles led to a number of 2,797 eligible references, which were classified into the categories ‘clinical references’ (243), ‘*in vivo* references’ (428), ‘*in vitro* references’ (1258), ‘pharmacognostic references’ (704) and ‘review references’ (164). The terminal screening of the abstracts of all clinical, *in vivo* and *in vitro* references revealed a final number of 418 references (77 clinical, 84 *in vivo*, 258 *in vitro*) (Additional file 3). Due to the fact that more than one reference could be defined from some studies, the systematic literature research led to a number of 378 studies representing the effects and efficacy of 29 plant species in 418 references. For one plant species, *Quercus robur* L., no references were found according to the criteria.

**Table 2:** Quantifying and categorizing of scientific publications regarding 30 medicinal plants during bibliographic literature research process

Plant species	Common name	All references imported from WoS^1^ and PM^2^after removal of duplicates	After keyword search in titles and abstracts with endnote	Pharmacognostic studies	Reviews	After checking of relevance, regarded for the assessment of plant species	Clinical studies	*In vivo* studies	*In vitro* and *ex vivo* studies
*Achillea millefolium* L.	yarrow	345	157	14	2	15	2	3	10
*Agrimonia eupatoria* L.	agrimony	73	37	10	0	2	1	0	1
*Allium sativum* L.	garlic	1149	630	24	14	31	9	5	17
*Althaea officinalis* L.	marshmellow	62	29	6	1	6	0	4	2
*Camellia sinensis* (L). KUNTZE	green tea	2052	804	53	21	32	4	6	22
*Carum carvi* L.	caraway	191	95	28	6	5	0	1	4
*Cetraria islandica* (L.) ACH.	Icelandic moss	118	46	8	0	4	1	1	2
*Echinacea purpurea* (L.) MOENCH	purple coneflower	869	364	45	18	48	14	8	26
*Foeniculum vulgare* (L.) MILL.	fennel	825	308	46	6	18	1	4	13
*Glycyrrhiza glabra* L.	liquorice	597	252	43	8	26	3	7	16
*Linum usitatissimum* L.	linseed	762	227	12	5	7	3	3	1
*Malva sylvestris* L.	wild mallow	243	50	2	2	4	0	3	1
*Matricaria recutita* L.	camomile	908	305	43	7	22	1	7	14
*Mentha x piperita* L.	peppermint	1331	425	35	23	21	8	0	13
*Origanum vulgare* L.	oregano	904	526	42	0	36	10	4	22
*Picea abies* (L.) H.KARST.	norway spruce	1031	153	0	0	1	0	0	1
*Pimpinella anisum* L.	anis	453	147	12	2	12	2	0	10
*Plantago lanceolata* L.	english plantain	532	136	7	2	6	1	0	5
*Potentilla erecta* (L.) RAEUSCH.	tormentil	49	15	3	2	3	2	0	1
*Primula veris* L.	cowslip	106	23	2	0	1	0	0	1
*Quercus robur* L.	english oak	1210	165	7	0	0	0	0	0
*Rubus fruticosus* L.	blackberry	583	172	34	0	6	0	1	5
*Rumex* ssp. L.	dock	939	208	15	2	11	0	4	7
*Salix* ssp. L.	willow	915	171	20	13	6	0	1	5
*Salvia officinalis* L.	sage	902	372	67	5	20	7	7	6
*Sambucus nigra* L.	elderberry	891	169	19	4	7	1	2	4
*Thymus vulgaris* L.	thyme	831	372	52	3	36	6	2	28
*Tussilago farfara* L.	coltsfoot	101	36	14	0	4	0	1	3
*Urtica dioica* L.	stinging nettle	760	251	16	10	20	1	6	13
*Vaccinium myrtillus* L*.*	blueberry	632	155	25	7	8	0	4	4
sum^a^		20364	6800	704	164	418	77	84	257

^a^due to the definition of reference (trial x plant species x indication) the sum may contain some trials more than one time; ^1^WoS = Web of Science [50]; ^2^PM = PubMed [49]

A total of 19,077 references were excluded because they did not match the predefined selection criteria. Predominant reasons for exclusion included that the content of title and abstract did not correspond to the focus of the review (e. g. pathogens were not the pathogens of the focused diseases). Other reasons were missing abstracts (in 212 references) or publications that were not peer-reviewed.

From the 418 remaining references, 48 references based on clinical studies were veterinary origin with 19 swine studies, 5 cattle studies, 17 horse studies, and 4 studies in rabbits. A number of 370 references include studies in humans (29 clinical, 84 *in vivo* and 257 *in vitro* studies). A number of 77 *in vivo* references used laboratory rodents (rats, mice, guinea-pigs) and three studies used cats as an animal model. For gastrointestinal indications (QA), 198 references were found, 57 references were related to respiratory diseases (QR), and 163 references aimed at the modulation of the immune system and inflammation processes (QL). Most references coping with the inclusion criteria were found for *Echinacea purpurea* L. MOENCH. (48 references), *Origanum vulgare* L. (36 references) *Thymus vulgaris* L. (36 references), *Camellia sinensis* (L.) KUNTZE (32 references), and *Allium sativum* L. (31 references). The required effects of a treatment and the proven effects of the plant species as mono-substances for each indication are shown in Tables 3, 4 and 5. In Table 6, the most promising plant species of the peer-reviewed references according to the scoring system for each indication (QA, QR and QL), as well as the most frequently recommended plant species of the traditional references (Additional file 2) are shown. According to the scoring system, the two most promising plant species are *Echinacea purpurea* (L.) MOENCH (for QR and QL) and *Allium sativum* L. (for QA).

**Table 3:** Assessment^a^ of medicinal plants based on peer-reviewed references^b^ of the last 20 years aiming gastrointestinal indications^c^

Plant species	Number of references	Type of reference	Antibacterial			Synergism with AB			Antiviral			Antiprotozoal			Antiadhesive			Antidiarrheal			Spasmolytic			Immunostimulant			Antiinflammatory			Analgesic			Hypothesis proved		
			**+**	**?**	**o**	**+**	**?**	**o**	**+**	**?**	**o**	**+**	**?**	**o**	**+**	**?**	**o**	**+**	**?**	**o**	**+**	**?**	**o**	**+**	**?**	**o**	**+**	**?**	**o**	**+**	**?**	**o**	**+**	**?**	**o**
*Achillea millefolium* L.	6	*in vitro*	2^1^																		4^2^														
	2	*in vivo + clinical*			1^3^																1^4^														
*Allium sativum* L.	12	*in vitro*	12^5^			1^6^																													
	9	*in vivo + clinical*	2^7^	1^8^								2^9^						2^10^						1^11^			1^12^						2^13^		
*Althaea officinalis* L.	2	*in vitro*	2^14^																																
	0	*in vivo + clinical*																																	
*Camellia sinensis* (L.) KUNTZE	8	*in vitro*	6^15^			1^16^											1^17^				1^18^														
	3	*in vivo + clinical*	2^19^															2^20^																	
*Carum carvi* L.	4	*in vitro*	3^21^																		1^22^														
	1	*in vivo + clinical*																									1^23^								
*Echinacea purpurea* (L.) MOENCH	0	*In vitro*																																	
	1	*in vivo + clinical*		1^24^																															
*Foeniculum vulgare* (L.) MILL.	10	*in vitro*	6^25^	1^26^	1^27^	1^28^								1^29^													1^30^								
	2	*in vivo + clinical*																			1^31^														1^32^
*Glycyrrhiza glabra* L.	9	*in vitro*	4^33^		2^34^				1^35^												1^36^						1^37^								
	4	*in vivo + clinical*	2^38^																		1^39^												1^40^		
*Linum usitatissimum* L.	0	*in vitro*																																	
	3	*in vivo + clinical*																1^41^			1^42^												1^43^		1^44^
*Matricaria recutita* L.	9	*in vitro*	5^45^																		4^46^														
	3	*in vivo + clinical*																2^47^															1^48^		
Plant species	Number of references	Type of reference	Antibacterial			Synergism with AB			Antiviral			Antiprotozoal			Antiadhesive			Antidiarrheal			Spasmolytic			Immunostimulant			Antiinflammatory			Analgesic			Hypothesis proved		
			**+**	**?**	**o**	**+**	**?**	**o**	**+**	**?**	**o**	**+**	**?**	**o**	**+**	**?**	**o**	**+**	**?**	**o**	**+**	**?**	**o**	**+**	**?**	**o**	**+**	**?**	**o**	**+**	**?**	**o**	**+**	**?**	**o**
*Mentha x piperita* L.	13	*in vitro*	8^49^	1^50^		1^51^															3^52^														
	7	*in vivo* + clinical	1^53^																1^54^		4^55^												1^56^		
*Origanum vulgare* L.	21	*in vitro*	18^57^	2^58^																							1^59^								
	8	*in vivo* + clinical	1^60^									2^61^						1^62^		1^63^						2^64^	1^65^						1^66^		
*Picea abies* (L.) H.KARST.	1	*in vitro*	1^67^																																
	0	*in vivo* + clinical																																	
*Pimpinella anisum* L.	7	*in vitro*	3^68^	2^69^		2^70^															1^71^														
	0	*in vivo* + clinical																																	
*Plantago lanceolata* L.	1	*in vitro*																			1^72^														
	1	*in vivo* + clinical			1^73^																														
*Potentilla erecta* (L.) RAEUSCH.	0	*in vitro*																																	
	2	*in vivo* + clinical													1^74^													1^75^							
*Rubus fruticosus* L.	1	*in vitro*										1^76^																							
	0	*in vivo* + clinical																																	
*Rumex ssp.* L.	4	*in vitro*	4^77^																																
	1	*in vivo* + clinical																1^78^																	
*Salix ssp.* L.	1	*in vitro*																			1^79^														
	0	*in vivo* + clinical																																	
*Salvia officinalis* L.	6	*in vitro*	5^80^	1^81^																															
	5	*in vivo* + clinical	3^82^									2^83^						1 ^84^	1^85^		1^86^			1^87^											
*Sambucus nigra* L.	1	*in vitro*	1^88^																																
	1	*in vivo* + clinical																									1^89^								
*Thymus vulgaris* L.	14	*in vitro*	10^90^	1^91^																	3^92^														
	3	*in vivo* + clinical			2^93^																			1^94^											
Plant species	Number of references	Type of reference	Antibacterial			Synergism with AB			Antiviral			Antiprotozoal			Antiadhesive			Antidiarrheal			Spasmolytic			Immunostimulant			Antiinflammatory			Analgesic			Hypothesis proved		
			**+**	**?**	**o**	**+**	**?**	**o**	**+**	**?**	**o**	**+**	**?**	**o**	**+**	**?**	**o**	**+**	**?**	**o**	**+**	**?**	**o**	**+**	**?**	**o**	**+**	**?**	**o**	**+**	**?**	**o**	**+**	**?**	**o**
*Tussilago farfara* L.	1	*in vitro*	1^95^																																
	0	*in vivo* + clinical																																	
*Urtica dioica* L.	6	*in vitro*	2^96^		2^97^				2^98^																										
	2	*in vivo* + clinical																									2^99^								
*Vaccinium myrtillus* L*.*	2	*in vitro*				1^100^						1^101^																							
	2	*in vivo* + clinical																								1^102^	1^103^		1^104^						
Sum of plant species for each effect		*in vitro*	18	6	3	6	0	0	2	0	0	2	0	1	0	0	1	0	0	0	10	0	0	0	0	0	3	0	0	0	0	0	0	0	0
		*in vivo* + clinical	6	2	3	0	0	0	0	0	0	3	0	0	1	0	0	7	2	1	6	0	0	3	0	2	6	1	1	0	0	0	6	0	2
Sum of assessments		*in vitro*	93	8	5	7	0	0	3	0	0	2	0	0	0	0	1	0	0	0	20	0	0	0	0	0	3	0	0	0	0	0	0	0	0
		*in vivo* + clinical	11	2	4	0	0	0	0	0	0	6	0	0	1	0	0	10	2	1	7	0	0	3	0	3	7	1	1	0	0	0	7	0	2

^a^Assessment = conclusion of a reference on a hypothesized effect; ^b^reference = trial x plant species x indication; ^c^particularly unspecified or infectious diarrhea and gastrointestinal spasms **+** = reference proves evidently the hypothesized effect; **?** = reference shows uncertain hypothesized effect; **o** = reference does not prove evidently the hypothesized effect

^1^[147, 148] ^2^[149–152] ^3^[153] ^4^[154] ^5^[69–76, 155–158] ^6^[69] ^7^[63, 66] ^8^[159] ^9^[62, 160] ^10^[63, 64] ^11^[66] ^12^[77] ^13^[161, 162] ^14^[163, 164] ^15^[165–170] ^16^[170] ^17^[171] ^18^[172] ^19^[124, 125] ^20^[124, 173] ^21^[174–176] ^22^[177] ^23^[178] ^24^[179] ^25^[175, 176, 180–183] ^26^[184] ^27^[185] ^28^[181] ^29^[186] ^30^[187] ^31^[188] ^32^[189] ^33^[143, 190–192] ^34^[142, 193] ^35^[145] ^36^[194] ^37^[195] ^38^[196, 197] ^39^[141] ^40^[198] ^41^[199] ^42^[199] ^43^[200] ^44^[201] ^45^[202] [175, 185, 203, 204] ^46^[205–208] ^47^[209, 210] ^48^[189] ^49^[176, 211–216] ^50^[217] ^51^[214] ^52^[88, 205, 218] ^53^[219] ^54^[220] ^55^[86, 87, 221, 222] ^56^[223] ^57^[52, 118, 175, 224–237] ^58^[238, 239] ^59^[240] ^60^[241] ^61^[242, 243] ^62^[244] ^63^[245] ^64^[244, 246] ^65^[247] ^66^[248] ^67^[249] ^68^[175, 250, 251] ^69^[252, 253] ^70^[251, 254] ^71^[255] ^72^[53] ^73^[256] ^74^[257] ^75^[258] ^76^[259] ^77^[180, 260–262] ^78^[263] ^79^[264] ^80^[175, 211, 233, 237, 265] ^81^[252] ^82^[266–268] ^83^[267, 268] ^84^[269] ^85^[270] ^86^[269] ^87^[268] ^88^[271] ^89^[272] 90[52, 118–120, 175, 176, 226, 250, 252, 273] ^91^[274] ^92^[113, 275, 276] ^93^[277, 278] ^94^[279] ^95^[280] ^96^[191, 281] ^97^[185, 282] ^98^[145] ^99^[283, 284] ^100^[285] ^101^[259] ^102^[286] ^103^[287] ^104^[286]

**Table 4:** Assessment^a^ of medicinal plants based on peer-reviewed references^b^ of the last 20 years aiming respiratory indications

Plant species	Number of references	Type of reference	Anti-bacterial			Synergism with AB			Antiviral			Spasmolytic			Expectorant			Antitussive			Immuno-stimulant			Anti-inflammatory			Analgesic			Hypothesis proved		
			**+**	**?**	**o**	**+**	**?**	**o**	**+**	**?**	**o**	**+**	**?**	**o**	**+**	**?**	**o**	**+**	**?**	**o**	**+**	**?**	**o**	**+**	**?**	**o**	**+**	**?**	**o**	**+**	**?**	**o**
*Achillea millefolium* L.	2	*in vitro*										2^1^																				
	1	*in vivo* + clinical										1^2^																				
*Agrimonia eupatoria* L.	1	*in vitro*							1^3^																							
	0	*in vivo* + clinical																														
*Allium sativum* L.	1	*in vitro*										1^4^																				
	0	*in vivo* + clinical																														
*Althaea officinalis* L.	0	*in vitro*																														
	4	*in vivo* + clinical																4^5^														
*Camellia sinensis* (L). KUNTZE	0	*in vitro*																														
	2	*in vivo* + clinical	1^6^					1^7^							1^8^																	
*Cetraria islandica* (L.) ACH.	0	*in vitro*																														
	1	*in vivo* + clinical																						1^9^								
*Echinacea purpurea* (L.) MOENCH	3	*in vitro*							2^10^															2^11^								
	8	*in vivo* + clinical	1^12^						1^13^		1^14^										2^15^		1^16^							2^17^		2^18^
*Foeniculum vulgare* (L.) MILL.	2	*in vitro*										2^19^																				
	0	*in vivo* + clinical																														
*Glycyrrhiza glabra* L.	1	*in vitro*							1^20^																							
	2	*in vivo* + clinical										1^21^						1^22^														
*Mentha x piperita* L.	1	*in vitro*										1^23^																				
	0	*in vivo* + clinical																														
*Pimpinella anisum* L.	2	*in vitro*										2^24^																				
	0	*in vivo* + clinical																														
*Plantago lanceolata* L.	1	*in vitro*										1^25^																				
	0	*in vivo* + clinical																														
*Primula veris* L.	1	*in vitro*	1^26^																													
	0	*in vivo* + clinical																														
*Rubus fruticosus* L.	0	*in vitro*																														
	1	*in vivo* + clinical																						1^27^								
*Rumex ssp.* L.	2	*in vitro*	1^28^						2^29^																							
	0	*in vivo* + clinical																														
*Salvia officinalis* L.	0	*in vitro*																														
	2	*in vivo* + clinical																1^30^									1^31^					
*Sambucus nigra* L.	1	*in vitro*							1^32^																							
	1	*in vivo* + clinical																												1^33^		
*Thymus vulgaris* L.	9	*in vitro*	3^34^									6^35^																				
	1	*in vivo* + clinical																												1^36^		
*Tussilago farfara* L.	1	*in vitro*	1^37^																													
	1	*in vivo* + clinical													1^38^			1^39^														
*Urtica dioica* L.	3	*in vitro*	3^40^																													
	1	*in vivo* + clinical							1^41^																							
*Vaccinium myrtillus* L*.*	1	*in vitro*							1^42^																							
	0	*in vivo* + clinical																														
Sum of plant species for each effect		*in vitro*	5	0	0	0	0	0	6	0	0	7	0	0	0	0	0	0	0	0	0	0	1	1	0	0	0	0	0	0	0	0
		*in vivo* + clinical	2	0	0	0	0	1	2	0	1	2	0	0	2	0	0	4	0	0	1	0	0	2	0	0	1	0	0	3	0	1
Sum of assessments		*in vitro*	9	0	0	0	0	0	8	0	0	15	0	0	0	0	0	0	0	0	0	0	1	2	0	0	0	0	0	0	0	0
		*in vivo* + clinical	2	0	0	0	0	1	2	0	1	2	0	0	2	0	0	7	0	0	2	0	0	2	0	0	1	0	0	4	0	2

^a^ Assessment = conclusion of a reference on a hypothesized effect; ^b^ reference = trial x plant species x indication; **+** = reference proves evidently the hypothesized effect; **?** = reference shows uncertain hypothesized effect; **o** = reference does not prove evidently the hypothesized effect

^1^ [288, 289] ^2^ [290] ^3^ [291] ^4^ [292] ^5^ [293–296] ^6^ [297] ^7^ [298] ^8^ [297] ^9^ [299] ^10^ [300, 301] ^11^ [103, 301] ^12^ [302] ^13^ [94] ^14^ [96] ^15^ [302, 303] ^16^ [96] ^17^ [95, 304] ^18^ [305, 306] ^19^ [307, 308] ^20^ [309] ^21^ [140] ^22^ [139] ^23^ [89] ^24^ [310, 311] ^25^ [53] ^26^ [312] ^27^ [313] ^28^ [314] ^29^ [315] ^30^ [293] ^31^ [316] ^32^ [300] ^33^ [317] ^34^ [191, 318, 319] ^35^ [113–116, 320, 321] ^36^ [108] ^37^ [322] ^38^ [323] ^39^ [323] ^40^ [322, 324, 325] ^41^ [326] ^42^ [327]

**Table 5:** Assessment^a^ of medicinal plants based on peer-reviewed references^b^ of the last 20 years aiming the modulation of the immune system and inflammation

Plant species	Number of references	Type of reference	Immunostimulant			Antiinflammatory			Analgesic			Hypothesis proved		
			**+**	**?**	**o**	**+**	**?**	**o**	**+**	**?**	**o**	**+**	**?**	**o**
*Achillea millefolium* L.	2	*in vitro*				2^1^								
	2	*in vivo* + clinical			1^2^			1^3^			1^4^			
*Agrimonia eupatoria* L.	0	*in vitro*												
	1	*in vivo* + clinical				1^5^								
*Allium sativum* L.	4	*in vitro*	1^6^	1^7^		2^8^								
	5	*in vivo* + clinical	5^9^			1^10^								
*Camellia sinensis* (L). KUNTZE	14	*in vitro*	3^11^			12^12^								
	5	*in vivo* + clinical	2^13^			2^14^			1^15^					
Cetraria islandica (L.) ACH.	2	*in vitro*	2^16^											
	1	*in vivo* + clinical				1^17^								
*Echinacea purpurea* (L.) MOENCH	23	*in vitro*	16^18^	1^19^		6^20^								
	13	*in vivo* + clinical	9^21^		2^22^							1^23^		1^24^
*Foeniculum vulgare* (L.) MILL.	1	*in vitro*				1^25^								
	3	*in vivo* + clinical				1^26^			3^27^					
*Glycyrrhiza glabra* L.	6	*in vitro*	4^28^			2^29^								
	4	*in vivo* + clinical	3^30^			1^31^			1^32^					
*Linum usita-tissimum* L.	1	*in vitro*				1^33^								
	3	*in vivo* + clinical		1^34^		2^35^			1^36^					
*Malva sylvestris* L.	1	*in vitro*				1^37^								
	3	*in vivo* + clinical	1^38^			2^39^			1^40^					
*Matricaria recutita* L.	5	*in vitro*		1^41^		4^42^								
	5	*in vivo* + clinical	1^43^		1^44^	2^45^			2^46^					
*Mentha x piperita* L.	0	*in vitro*												
	1	*in vivo* + clinical							1^47^					
*Origanum vulgare* L.	1	*in vitro*				1^48^								
	6	*in vivo* + clinical	2^49^						3^50^				1^51^	
*Pimpinella anisum* L.	1	*in vitro*				1^52^								
	2	*in vivo* + clinical	1^53^	1^54^										
*Plantago lanceolata* L.	3	*in vitro*				3^55^								
	0	*in vivo* + clinical												
*Potentilla erecta* (L.) RAEUSCH.	1	*in vitro*				1^56^								
	0	*in vivo* + clinical												
*Rubus fruticosus* L.	4	*in vitro*				4^57^								
	0	*in vivo* + clinical												
*Rumex ssp.* L.	1	*in vitro*				1^58^								
	3	*in vivo* + clinical				2^59^			2^60^					
*Salix ssp.* L.	4	*in vitro*				4^61^								
	1	*in vivo* + clinical				1^62^								
*Salvia officinalis* L.	0	*in vitro*												
	7	*in vivo* + clinical	1^63^	1^64^	1^65^	4^66^			2^67^					
*Sambucus nigra* L.	2	*in vitro*	1^68^			1^69^								
	1	*in vivo* + clinical			1^70^									
*Thymus vulgaris* L.	5	*in vitro*				5^71^								
	4	*in vivo* + clinical		1^72^	1^73^				2^74^					
*Tussilago farfara* L.	1	*in vitro*				1^75^								
	0	*in vivo* + clinical												
*Urtica dioica* L.	4	*In vitro*	2^76^			2^77^								
	4	*in vivo* + clinical	3^78^			1^79^			1^80^					
*Vaccinium myrtillus* L*.*	1	*In vitro*				1^81^								
	2	*in vivo* + clinical	1^82^			2^83^								
Sum of plant species for each effect		*in vitro*	6	3	0	20	0	0	0	0	0	0	0	0
		*in vivo* + clinical	9	4	6	13	0	1	12	0	1	1	1	1
Sum of assessments		*in vitro*	29	3	0	56	0	0	0	0	0	0	0	0
		*in vivo* + clinical	24	4	7	20	0	1	20	0	1	1	1	1

^a^Assessment = conclusion of a reference on a hypothesized effect; ^b^reference = trial x plant species x indication; **+** = reference proves evidently the hypothesized effect; **?** = reference shows uncertain hypothesized effect; **o** = reference does not prove evidently the hypothesized effect

^1^[328, 329] ^2^[330] ^3^[331] ^4^[331] ^5^[332] ^6^[333] ^7^[334] ^8^[78, 80] ^9^[65, 67, 68, 79, 335] ^10^[79] ^11^[133, 336, 337] ^12^[336, 338–348] ^13^[134, 349] ^14^[131, 350] ^15^[351] ^16^[352, 353] ^17^[354] ^18^[99, 100, 355–368] ^19^[369] ^20^[102, 370–374] ^21^[93, 97, 98, 375–380] ^22^[381, 382] ^23^[383] ^24^[384] ^25^[385] ^26^[386] ^27^[386–388] ^28^[144, 334, 389, 390] ^29^[391, 392] ^30^[138, 380, 393] ^31^[394] ^32^[394] ^33^[395] ^34^[396] ^35^[397, 398] ^36^[397] ^37^[399] ^38^[400] ^39^[401, 402] ^40^[401] ^41^[403] ^42^[404–407] ^43^[408] ^44^[409] ^45^[410, 411] ^46^[410, 412] ^47^[413] ^48^[414] ^49^[415, 416] ^50^[417–419] ^51^[420] ^52^[421] ^53^[422] ^54^[423] ^55^[392, 424, 425] ^56^[426] ^57^[427–430] ^58^[431] ^59^[432, 433] ^60^[432, 434] ^61^[426, 435–437] ^62^[438] ^63^[439] ^64^[440] ^65^[441] ^66^[442–445] ^67^[442, 443] ^68^[446] ^69^[430] ^70^[447] ^71^[436, 448–450] ^72^[451] ^73^[451] ^74^[417, 452] ^75^[453] ^76^[454, 455] ^77^[456, 457] ^78^[138, 458, 459] ^79^[460] ^80^[460] ^81^[344] ^82^[461] ^83^[461, 462]

**Table 6:** Promising medicinal plants for treatment and prophylaxis of gastrointestinal and respiratory diseases and for modulation of the immune system and inflammation

Indication	Traditional applications in *initial sources* ^*1*^ (number of references recommending the plant for the indication)^a^	Peer-reviewed references (sum of points gathered in the scoring system^2^)^b^
Gastrointestinal tract	***Matricaria recutita*** **L**. (7)	*Allium sativum* L. (41)
	***Foeniculum vulgare*** **(L.) MILL.** (6)	***Mentha x piperita*** **L.** (30)
	*Potentilla erecta* (L.) RAEUSCH (5)	***Salvia officinalis*** **L.** (27)
	*Linum usitatissimum* L. (5)	*Origanum vulgare* L. (24)
	*Rubus fruticosus* L. (5)	***Camellia sinensis*** **(L.) KUNTZE** (18)
	***Thymus vulgaris*** **L.** (4)	***Matricaria recutita*** **L.** (15)
	*Quercus robur* L. (4)	*Glycyrrhiza glabra* L. (14)
	***Mentha x piperita*** **L.** (4)	***Thymus vulgaris*** **L.** (13)
	***Urtica dioica*** **L.** (4)	***Foeniculum vulgare*** **(L.) MILL.** (7)
	*Vaccinium myrtillus* L. (4)	***Carum carvi*** **L.** (6)
	***Salvia officinalis*** **L.** (4)	*Pimpinella anisum* L. (6)
	***Carum carvi*** **L.** (4)	*Rumex sp.* L. (6)
	***Camellia sinensis*** **(L.) KUNTZE** (4)	***Urtica dioica*** **L.** (6)
	*Achillea millefolium* L. (4)	
Respiratory tract	***Thymus vulgaris*** **L.** (7)	*Echinacea purpurea* (L.) MOENCH (10)
	*Pimpinella anisum* L. (6)	***Thymus vulgaris*** **L.** (10)
	***Althaea officinalis*** **L.** (5)	***Althaea officinalis*** **L.** (8)
	*Cetraria islandica* (L.) ACH. (5)	*Glycyrrhiza glabra* L. (5)
	*Primula veris* L. (5)	*Salvia officinalis* L. (5)
	*Foeniculum vulgare* (L.) MILL. (4)	*Tussilago farfara L.* (5)
	***Sambucus nigra*** **L.** (4)	*Urtica dioica* L. (5)
	*Malva sylvestris* L. (4)	*Achillea millefolium* L. (4)
	*Allium sativum* L. (4)	*Camellia sinensis* (L.) KUNTZE (4)
	*Picea abies* (L.) H.KARST. (4)	***Sambucus nigra*** **L.** (4)
Modulation of immune system and inflammation	***Echinacea purpurea*** **(L.) MOENCH** (2)	***Echinacea purpurea*** **(L.) MOENCH** (41)
	*Salix* sp. L. (2)	*Camellia sinensis* (L.) KUNTZE (26)
	*Thymus vulgaris* L. (1)	*Glycyrrhiza glabra* L. (19)
	*Sambucus nigra* L. (1)	*Origanum vulgare* L. (19)
	*Urtica dioica* L. (1)	***Allium sativum*** **L.** (18)
	***Malva sylvestris*** **L.** (1)	*Salvia officinalis* L. (16)
	*Plantago lanceolata* L. (1)	*Urtica dioica* L. (15)
	***Allium sativum*** **L.** (1)	*Foeniculum vulgare* (L.) MILL. (11)
	*Tilia cordata* MILL/*Tilia platyphyllos* SCOP. (1)	*Matricaria recutita* L. (11)
	*Artemisia absynthum* L. (1)	***Malva sylvestris*** **L** ***.*** (9)
	*Verbascum* sp. L. (1)	*Rumex* sp. L. (9)
	*Armoracia rusticana* PH. GÄRTN*.(1)*	

^1^initial sources = standard literature, based on traditional empiric knowledge and historical literature of veterinary [42–45], and human phytotherapy [46], peer-reviewed publications of European [47] and Swiss ethnoveterinary medicine [36, 38] and a report of the European Food Safety Authority (EFSA) [48] focusing on the use of plants as feed additives in animal production; ^2^Score = 3 x (number of proven effects in clinical studies – number disproof of effects in clinical studies) + 2 x (number of proven effects in *in vivo* studies - number of disproof of effects in *in vivo* studies) + 1 x (number of proven effects in *in vitro* studies - number of disproof of effects of *in vitro* studies) ^a^gastrointestinal tract^:^ all plant species recommended for QA 4 times or more in the initial *sources*, ordered by incidence of recommendation by different authors as listed in detail in Additional file 2; ^a^respiratory tract: all plant species recommended for QR 4 times or more in the *initial sources*, ordered by incidence of recommendation by different authors as listed in detail in Additional file 2; ^a^Modulation of immune system and inflammation: includes all plant species recommended for QL 1 time or more in the *initial* sources, ordered by incidence of recommendation by different authors as presented in file 2 in greater detail; ^b^gastrointestinal tract: includes all plant species gathered for QA with a minimum score of 6 ordered by sum of points the plant species gathered; ^b^respiratory tract: includes all plant species gathered for QR with a minimum score of 4, ordered by sum of points the plant species gathered; ^b^Modulation of immune system and inflammation: includes all plant species gathered for QL with a minimum score of 9, ordered by sum of points the plant species gathered; **bold letters** = plant species recommended in *initial sources* and in peer-reviewed references for same indication

## Discussion

There is a large amount of evidence-based knowledge about medicinal plants, represented by 20,364 studies focusing on 30 medicinal plant species from the last 20 years considering peer-reviewed publications in English or German language. The emergence of multi-drug resistance in human and animal pathogens results in international accordance to strengthen the research in novel treatment options. Medicinal plants and their extracts might be an option to prevent and cure livestock diseases.

### Evaluation of the search strategy

This systematic review was designed and performed according to the guidelines of the PRISMA statement and AMSTAR measurement tool [39–41]. Due to the fact that we searched for available data on a largely underrepresented topic in the last decades, only a small number of veterinary clinical data is currently available. Therefore, the search strategy was adapted to gain as much plant specific information as possible and to cope with the complex research question. Human clinical studies, experimental *in vivo* studies with laboratory animals as well as *ex vivo* and *in vitro* studies were included as well. To avoid the risk of source selection bias, multiple types of sources were used initially: standard textbooks, peer-reviewed publications, a governmental report, and personal communications with experts. The risk of introducing database bias was reduced by using two different and independent databases and by using the Mesh Terms function of PubMed. The selection of the 30 traditionally used plant species may bear a sampling bias. European ethnoveterinary and traditional administrations of medicinal plants were screened to identify promising plant species for the bibliographic search. Due to our strategy, it is likely that frequently studied plant species come up as more promising compared to less frequently studied plants. Additionally, the timeliness of the review excluded studies published before 1994 which may be accepted in science for decades by e.g. the European Scientific Cooperative on Phytotherapy [55]. As a consequence, plants including *Malva sylvestris* L., *Potentilla erecta* (L.) RAEUSCH, *Primula veris* L., *Quercus robur* L. or *Picea abies* (L.) H.KARST. appeared less promising, although they are an integral element of traditional medicine.

### Comparison of traditional phytotherapy with up-to-date knowledge

The most promising plant species of the peer-reviewed publications of the last 20 years were compared to the most common traditional administrations of the *initial sources* (Table 6). For the most promising plant species 62, 30 and 27 % are also frequently recommended in the *initial sources* for QA, QR and QL, respectively. The results confirmed the rationale of some traditional administrations of medicinal plants. Cases where the traditional applications were not confirmed by current studies, may be explained by the fact that only studies published between 1994 and 2014 were considered. Nevertheless, for many of these plant species broad scientific substantiation exists. For example, ESCOP monographs are available for *Linum usitatissimum* L., *Pimpinella anisum* L., *Cetraria islandica* (L.) ACH., *Primula veris* L. and *Salix* ssp..

### Complexity of varying chemistry

It is important to consider that the amount of active constituents in plants depends on environmental factors. Based on the plant cultivars used climatic and geographic conditions, quality of the soil and method of cultivation and harvest influence the phytochemical composition of the plant and therefore, different amounts of constituents can be found in different batches. The used part of plant, widely divergent post-harvesting processing and methods of extraction and stabilization affect the chemistry of phytopharmaceuticals [56]. Environmental factors and post harvesting procedures are likely to explain varying effectiveness of a medicinal plant in different studies as reported for Echinacea [57, 58]. Therefore, a direct comparison of the outcome of the studies is difficult especially because of the lack of information regarding the phytochemical composition of the used test material. The use of pharmacopoeia quality in future research would ensure a defined amount of active constituents [59].

### Relevance for the treatment of livestock diseases

From the 418 references assessed, 46 focused on livestock including 19 clinical references for pigs and five for cattle. Most of these clinical studies used the plants as a feed additive and not as a pharmaceutical. This might be due to complex regulatory affairs exacerbating the licensing and authorization of a medicinal plant or a plant extract as a veterinary drug.

Due to missing information regarding the absorption of orally administered medicinal plant compounds by the gastrointestinal tract, local treatment of gastrointestinal diseases might promising compared to a systemic treatment of respiratory diseases. Effective concentrations of, e.g. essential oils via inhalation may be obtained in the respiratory tract, but it is less practicable when larger herds need to be treated. While pigs as monogastrics might be compared with humans, calves are young ruminants and the biotransformation of plants secondary metabolites in the forestomach is not well known. However, in suckling calves plant extracts can be administered by daily milk diet to ensure bypassing the forestomach by oesophagal groove reflex.

We identified only a few recent references and also few traditional recommendations for the indication QR (*n* = 57) compared to QA (*n* = 198). Interestingly, similar findings were reported from ethnoveterinary research [47, 60]. One explanation might include the challenge of the treatment of respiratory diseases because systemic effects are needed to obtain the therapeutic effect compared to primarily local effects. Modulation of the immature or deficient immune system of calves and piglets provides a starting-point for the prevention of multifactorial infectious diseases. In the traditional phytotherapy literature, effects of medicinal plants on the immune system cannot be found frequently. This might be explained by the fact that immunology is a relatively young scientific field that developed rapidly in recent years. In human medicine, some immune stimulating preparations are already available on the market and therefore, a variety of studies is available. In contrast, for livestock, scientific knowledge is not transferred to practical use yet.

This review mainly focused on therapeutic options of medicinal plants. From this point of view, the relevance of possible toxicity, adverse effects or residues in livestock products remains open. Regarding safety aspects *Tussilago farfara* L. cannot be recommended as a therapeutic medicinal plant due to the presence of toxic pyrrolizidine alkaloids. Nevertheless, the majority of plant species in this review are consumed by humans as food, spices, luxury foodstuffs or as registered nutraceuticals and pharmaceuticals. If these plant species are safe for ingestion in humans, it might be legitimate to transfer these results to other mammalians with a comparable metabolism (herbivores and omnivores). Under these circumstances, risks for humans based on residues in products from food-producing animals should be neglectable. For herbivores and omnivores with a mainly plant-based ration, safety of the most medicinal plant species can be supposed. These species may cope with plant secondary metabolites in a similar way as humans [35].

### Promising plant species for gastrointestinal and respiratory diseases and for modulation of the immune system and inflammation

Several trials show the equivalence of plant-derived pharmaceuticals with synthetic ones, but nonetheless there are some trials showing the contrary. Based on the data presented in this review, *Allium sativum* L., *Mentha x piperita* L. and *Salvia officinalis* L. carry a high potential for treatment of gastrointestinal diseases (Table 3). *Echinacea purpurea* (L.) MOENCH, *Thymus vulgaris* L. and *Althaea officinalis* L. may be considered for the treatment of respiratory diseases (Table 4). Regarding the majority of positive results of studies evaluated, *Echinacea purpurea* (L.) MOENCH, *Camellia sinensis* (L.) KUNTZE and *Glycyrrhiza glabra* L. were found to stimulate the immune response (Table 5).

Traditionally, plant species with a high content of tanning agents are administered in diarrhea. *Allium sativum* L. does not contain tanning agents, but due to its antibacterial, antidiarrheal, anti-inflammatory and immunomodulatory effects, it may be used for prophylactic and acute treatment in diarrhea of calves and piglets. Eight *in vivo* and *clinical* studies were identified for *Allium sativum* L. proving these effects, and no studies disproving them. A trial conducted with neonatal calves showed that allicin, a main active compound of *Allium sativum* L., delayed the onset of diarrhea due to *Cryptosporidium parvum* [61]. Two clinical studies demonstrated antidiarrheal effects and a reduction of the fecal coliform count by *Allium sativum L..* There is also evidence for an improvement of performance in pre-ruminant calves [62, 63]. The immunomodulatory activity of *Allium sativum* L. in pigs [64, 65] and poultry [66, 67] may hold true in immunocompromised calves to support their immune defense. Its antibacterial effects on *Escherichia coli* and *Salmonella* ssp. *in vitro* suggest a high probability of antibacterial activity *in vivo* [68–75]. Nevertheless, more clinical studies are necessary to investigate antiinfective effects of *Allium sativum* L. in young farm animals. *Allium sativum* L. has been reported to exhibit anti-inflammatory activity in rats [76]. There is also mechanistic evidence for anti-inflammatory properties as well as immunostimulation showed in three in *vitro studies*, namely an inhibition of leucocyte migration [77], modulation of interleukin and interferon-gamma expression [78] and a suppression of nitrogen oxide production in macrophages [79]. An anti-inflammatory effect may be useful for the treatment of systemic inflammation processes often accompanied with diarrhea. Facing animal welfare and regarding efficient synthetic non-steroidal and steroidal anti-inflammatory agents, it is debatable whether there is a need for plant-derived alternatives. While synthetic non-steroidal and steroidal anti-inflammatory agents often produce considerable adverse effects including an inhibition of mucus production [80, 81], medicinal plants compass considerable adverse-effects because they contain several different active compounds which might reduce the potential of unwanted effects [82]. In *in vivo* tests for acute and chronic toxicity, the maximum tolerance dose and genotoxicity *Allium sativum* L. was demonstrated to be relatively safe if administered in therapeutic dosages [83] and if estimated for the animals metabolic body weight [84]. With respect to food quality, it must be assured that residues of *Allium sativum* L., responsible for the typical taste of this plant do not result in an altered taste of meat.

To reduce enteral spasm during diarrheal diseases, *Mentha x piperita* L. might be an efficient treatment option based on three clinical studies in humans, demonstrating efficient spasmolytic activity comparable to butylscopolamine [85–87]. The underlying mechanism includes inhibition of smooth muscle contractility through the block of calcium influx by menthol [88]. In traditional medicine, *Mentha x piperita* L. has been used in the therapy of respiratory diseases. Peppermint essential oil showed spasmolytic activity on rat trachea *ex vivo* [89]. But there are no clinical studies in veterinary medicine for *Mentha x piperita* L. in respiratory disease. No adverse effects have been reported for *Mentha x piperita* L. infusions or oral intake of leaves [90]. Excessive inhalation or local application of pure *Mentha x piperita* L. essential oil was shown to lead to hypersensitivity reactions [56]. Contraindications are severe hepatic damage and cholestasis [43].

Based on this review, the most prominent plant species for stimulation of the immune system is *Echinacea purpurea* (L.) MOENCH. The main constituents are polysaccharides, alkylamides, caffeic acid esters and polyacetylenes [91]. It has been used in therapy for stimulation of the immune system in human medicine, mainly for prevention of viral infections of the respiratory tract [92]. A total of 23 clinical and *in vivo* studies revealed multiple effects on the innate and acquired immune system. *Echinacea purpurea* (L.) MOENCH was shown to increase the immune response towards swine erysipelas vaccination in piglets [93], prevented enveloped virus infections in humans [94] and reduced symptom severity in naturally acquired upper respiratory tract infections in humans [95]. In contrast, seven clinical or *in vivo* studies reported the absence of the above mentioned effects. For example *Echinacea purpurea* (L.) MOENCH failed to enhance growth or to show any immunomodulatory effect in one study in pigs [96]. Reasons for these negative results may be due to a very small number of individuals, an improper dosage or study design. As mentioned above, the diversity of non-standardized *Echinacea* preparations with varying chemistry is likely to result in different findings. In some studies, the dosage was not reported, and therefore it was not possible to estimate how much plant material or drug equivalent was administered per day. Consequently, due to missing data final conclusions cannot be drawn. Nevertheless, eight *in vivo* studies reported modulations of immune system and blood cell count, and no studies were found disproving these effects. Different Echinacea species were found to increase the total number of white and red blood cells in mice [97] and horses [98]. Twenty-six *in vitro* studies demonstrated the underlying mechanisms of immunomodulatory effects of *Echinacea purpurea* (L.) MOENCH. It was reported to activate macrophages and natural killer cells [99–101] and to modulate several cytokines [102–105]. *Echinacea purpurea* (L.) MOENCH is known as a safe immunostimulant in humans and several products are available on the market. No reported interactions with other drugs and no toxic effects after overdosage were reported [106]. Possible rare adverse effects such as hypersensitivity reactions are reported, but no adverse effects have been observed during long-term administration [107]. In general, *Echinacea purpurea* (L.) MOENCH seems to be effective in preventing respiratory diseases and as an early intervention immediately after onset of first symptoms of infectious diseases [95]. However, further veterinary clinical studies need to be performed, especially to evaluate effective dosages.

In human medicine, *Thymus vulgaris* L. has been already effectively used according to its antitussive and mucolytic effects in the treatment of acute bronchitis, often in combinations with other plant species, e. g. *Primula veris* L. for its expectorant effects [108–110]. The main active compound of *Thymus vulgaris* L. is the essential oil containing thymol, geraniol, thujanol and linalool [111]. The above mentioned effects still have to be investigated for veterinary purposes. Nevertheless, an enhancement of the mucociliary clearance in mice was shown in two *in vivo* studies [112, 113]. This effect was explained by an interaction with beta_2_ receptors in rat lung tissue [112]. Additionally, three *ex vivo* studies demonstrated spasmolytic effects of *Thymus vulgaris* L. on tracheal chains comparable to theophylline [114–116]. The reported anti-inflammatory properties [117] and antibacterial effects found in *in vitro* studies [118–120] of *Thymus vulgaris* L. still needs to be investigated in clinical studies. *In vivo* studies on the toxicology of *Thymus vulgaris* L. leaf extract showed no toxic potential [121, 122]. In summary, the data available support the potential for using *Thymus vulgaris* L. for treatment of respiratory diseases in livestock.

*Camellia sinensis* (L.) KUNTZE seems to be useful for treatment of diarrhea as well as for stimulation of the immune system. Main constituents are polyphenolic compounds (up to 25 % catechin derivatives in non-fermented plants, e.g. epigallocatechin), purine alkaloids (caffeine, theobromine, theophylline) and flavonoids [123]. Some clinical studies reported beneficial effects of *Camellia sinensis* (L.) KUNTZE on gut health as indicated by a reduced prevalence of postweaning diarrhea in piglets, but also a decrease in growth performance [124]. An experimental trial on a diet with *Camellia sinensis* (L.) KUNTZE whole plant extract revealed a significant decrease of *Clostridia* counts, but also of *Enterococci* counts in the feces of piglets compared to a standard diet with antibiotics [125]. Two *in vivo* studies showed also anti-influenza virus activity in mice [126] and chicken [127], which might be due to an inhibition of virus adsorption [126]. Fifteen *in vitro* and five *in vivo* studies demonstrated antioxidative [128–130] and anti-inflammatory [131, 132] effects and a modulation of the immune system [133, 134]. No studies were found disproving these effects. In mice, the intake of a concentrated extract of *Camellia sinensis* (L.) KUNTZE did not lead to unwanted adverse effects [135]. Despite that *Camellia sinensis* (L.) KUNTZE is known to be fairly devoid of unwanted effects, some reports on liver damage related to the intake of *Camellia sinensis* (L.) KUNTZE extract are available [136, 137]. In summary, most reports state that safety of *Camellia sinensis* (L.) KUNTZE extract can be supposed, if used appropriately to the recommendations [136]. Therefore, *Camellia sinensis* (L.) KUNTZE bears a reliable potential for prophylaxis and therapy of diseases in calves and piglets.

The main active compound of medicinally used roots of *Glycyrrhiza glabra* L. is the saponin glycyrrhizin. Furthermore, it contains flavonoids and isoflavonoids, chalcones, cumarins and phytosterols [123]. With regard to the inclusion criteria of this review, no clinical studies focusing on *Glycyrrhiza glabra* L. could be found. Nevertheless, it was shown to exhibit immunostimulatory effects *in vivo*, by stimulation of cellular and nonspecific response [138]. In three *in vivo* and *ex vivo* models, antitussive [139] and tracheal smooth muscle relaxing activity [140] as well as regulating effects in the gastrointestinal tract were reported [141]. Four *in vitro* studies demonstrated its antimicrobial [142, 143] and antiviral potential by activation of autophagy [144]. In an assessment of different plant species, *Glycyrrhiza glabra* L. exerted the strongest antiviral activity against rotavirus [145]. Due to these versatile effects, *Glycyrrhiza glabra* L. might be beneficial for prophylaxis and treatment of virus induced diseases of the respiratory- as well as the gastrointestinal tract. Regarding the safety of *Glycyrrhiza glabra* L., it is important to choose the right dosage due to the hyper-mineralocorticoid-like effects of glycyrrhizin. An acceptable daily dosage of 0.015–0.229 mg glycyrrhizin/kg body weight/day for human and animals was reported [146].

## Conclusions

This systematic review identified common medicinal plant species as a potential future therapeutic option for gastrointestinal and respiratory diseases in calves and piglets. Based on their plant specific multi-component compositions, the versatile effects of medicinal plants as ‘multi-target drugs’ may bear a potential for the treatment of respiratory and gastrointestinal diseases in calves and piglets. Medicinal plants are unlikely to replace chemical medications as a general rule, but they may be a single or at least a complementary treatment. In concert with housing, feeding and hygiene, medicinal plants are part of a sustainable, natural option for improving animal health and reducing the use of antimicrobials in livestock farming. The results of this review provide support for a need for additional *in vitro*, *in vivo* and clinical research focused on phytotherapy for recently emerging and challenging diseases in livestock. While a large amount of peer-reviewed studies about medicinal plant species is available, most of the clinical and experimental studies were performed in humans and experimental animals. More research is needed to evaluate the potential of medicinal plants for treatment of farm animals. The data from this review provide guidance on medicinal plants promising for further investigations in livestock: the most promising candidates for gastrointestinal diseases are *Allium sativum* L., *Mentha x piperita* L. and *Salvia officinalis* L.; for diseases of the respiratory tract, *Echinacea purpurea* (L.) MOENCH, *Thymus vulgaris* L. and *Althea officinalis* L. were found most promising, and *Echinacea purpurea* (L.) MOENCH, *Camellia sinensis* (L.) KUNTZE, *Glycyrrhiza glabra* L. and *Origanum vulgare* L. were identified as best candidates for modulation of the immune system and inflammation. Based on this review, studies are under way to investigate the effects of promising medicinal plants in calves and piglets.

## Abbreviations

ATCvet, Anatomical Therapeutic Chemical classification system for veterinary medicine; e.g., *exempli gratia* (in English: for example); EFSA, European Food Safety Authority; EMA, European Medicines Agency; ESCOP, European Scientific Cooperative On Phytotherapy; MBW, metabolic body weight; pH, pondus Hydrogenii; PICOS, population, intervention, comparator, outcome, study design; QA, preparations used for the treatment of diseases affecting the alimentary tract or metabolism, particularly unspecified or infectious diarrhea and intestinal spasms; QL, immunomodulating agents; QR, preparations for the treatment of diseases in the respiratory system; sp., any species, not specified in detail; ssp., subspecies; WHO, World Health Organization

## Additional files

Additional file 1: Protocol of the systematic review. (DOCX 27 kb) Additional file 2: Plant species recommended for the treatment of gastrointestinal and respiratory diseases in human and animals based on standard textbooks, European peer-reviewed ethnoveterinary publications and an EFSA report. (XLSX 42 kb) Additional file 3: Assessment of medicinal plants effects in 418 final peer-reviewed references. (XLSX 59 kb)
