# Supplementary material for: Medicinal plants – prophylactic and therapeutic options for gastrointestinal and respiratory diseases in calves and piglets? A systematic review
Source: BMC Vet Res. 2016 Jun 6;12:89. doi: 10.1186/s12917-016-0714-8 (PMC4896019; doi:10.1186/s12917-016-0714-8)
Supplement: Additional file 1: — Protocol of the systematic review. (DOCX 27 kb) [file 12917_2016_714_MOESM1_ESM.docx]

Medicinal plants – prophylactic and therapeutic options for gastrointestinal and respiratory diseases in calves and piglets? A systematic review

**Additional file 1: Protocol of the systematic review**

Aim of the systematic review:

The aim of this systematic literature review is to identify European medicinal plant species or their extracts that are promising candidates for use in infectious diseases of the gastrointestinal and respiratory tract in calves and piglets. Candidate plant species should bear a reliable potential for economic and effective treatment and prevention of these diseases. The result of this review can build a basis for state-of-the-art experimental trials and clinical studies.

Procedure:

1. Which are the most common diseases of youngstock?

Preparation of a survey over the most common diseases of the gastro-intestinal and respiratory tract of calves and piglets with using relevant textbooks, current peer-reviewed publications and expert knowledge. We excluded state-controlled infectious diseases in Switzerland and notifiable infectious diseases in Germany.

2. Which plant species are frequently recommended for the treatment of these diseases?

2.1. Screening of relevant *initial sources* based on primary and secondary literature

- standard literature of veterinary phytotherapy

- one textbook for phytotherapy of human medicine

- three current peer-reviewed publications of ethnoveterinary research

- a report of the EFSA about the use of plants as feed additives in animal production

2.2. Registering of all plant species in a table which are mentioned for the treatment of at least one of the following disease complexes

- gastrointestinal tract (GIT) diseases including the following indications: infectious diarrhea, unspecific diarrhea, infection of GIT, gastrointestinal spasms

- respiratory tract (RT) diseases including the following indications: cough, cold, infections of RT, pneumonia, bronchitis, broncho-pneumonia, rhinitis, laryngitis, tracheitis, pharyngitis, sinusitis, mucous obstruction of RT

- immunodeficiency and inflammation

2.3. Collecting data of these plant species concerning

- used part of plant

- indication, classified according to the ATCvet code system:

QA = preparations used for the treatment of diseases affecting the alimentary tract, particularly diarrhea and intestinal spasms

QR = preparations for the treatment of diseases in the respiratory system

QL = immunomodulating agents (and agents for modulation of inflammatory processes)

3. Which of the plant species detected are recommended most frequently in general and for each apparatus?

A preliminary selection of plant species which are mentioned at least in three of the reviewed books or publications for one of the three indications (QA, QR, QL) and thereof

- at least three times for the indication QA or QR or

- at least two times for the indication QL

is established.

4. Asking of experts in phytotherapy

This selection is sent to three specialists in European veterinary phytotherapy to capitalise their experience. The experts are asked to comment on the preliminary selection of plant species regarding the most common ones being particularly suitable for treatment and prevention of gastrointestinal and respiratory diseases.

5. Selection of plant species

Based on the preliminary selection of plant species and expert’s knowledge the final list of focus plant species is compiled. For these plant species, the literature search with online data bases is accomplished.

6. Searching for publications in online databases, based on an automatic search

6.1. Pub Med

6.1.1 Search with keywords

- name of the plant species in Latin and in English and the pharmaceutical name of the used part (e.g. “Foeniculum vulgare” OR “fennel” OR “foeniculi fructus”)

- only publications from 1994-01-01 until 2014 -12-31, written in English or German

- refining the results with “subjects”: complementary medicine, dietary supplements, systematic review, toxicology, veterinary science

- saving the publications in an EndNote-Data, for each plant in an own subfolder

6.1.2. Search with PubMed Mesh-Terms

- name of the plant species in Latin (e.g. “Foeniculum vulgare”)

- AND adverse effects, analysis, drug effects, microbiology, pharmacology, therapeutic use, therapy, toxicity

- only publications from 1994-1-1 until 2014-12-31, written only in English or German

- saving the publications in the EndNote-Data, for each plant in an own subfolder

6.2. Web of Knowledge

6.2.1. Search with keywords

- name of the plant species in Latin and in English and the pharmaceutical name of the used part: “Foeniculum vulgare” OR “fennel” OR “foeniculi fructus”

- only publications from until 1994-1-1 until 2014-12-31, written only in English or German

- refining the results with the research domain “science technology”

- refining the results with “research areas”: pharmacology, infectious diseases, toxicology, veterinary sciences, microbiology, gastroenterology, integrative complementary medicine, general internal medicine, respiratory system, virology

- saving the publications in the EndNote-Data, for each plant in an own subfolder.

7. Removing of duplicates for each plant with help of EndNote

The number of references for each plant is sampled. The used search terms and print screens of each search are stored.

8. Keyword search within EndNote

To refine the results, a term-list search is conducted within each plant species, including only references which contain one of the predefined keywords in their citation or abstract:

Anti* (e.g. antimicrobial, antibacterial, antiviral, antifungal, antinociceptive, anti-inflammatory…)

Immune*

Muco*

Spasmo*

Astring*

Pig*

Calv*

Wean*

Intest*

Gastro*

Pulmo*

Broncho*

Pharma*

Eff* (e.g. efficiency, effectivity, effect)

Bioactiv*

Constitu*

NOT: tumor

NOT: cancer

9. Sample check of excluded references

To avoid the exclusion of relevant references, the excluded ones are checked randomly.

10. Relevance screening – based on a manual procedure conducted through one person

Screening of the title (first step) and the abstract (second step) if the references suffice the claims measured by means of predefined inclusion and exclusion criteria. If references do not, they are excluded.

10.1. Inclusion criteria:

Inclusion of all references which

- provide an abstract
- are published in peer-reviewed journals
- investigate one single plant species, including different preparations like extracts or one single component of it

and dealing with

(a)

- an assessment of plants *in vitro*, *ex vivo,* *in vivo* or in clinical trials

AND

- antimicrobial/antibacterial/antiviral or antiprotozoal effects
- anti-inflammatory/analgesic effects
- antiadhesive effects
- astringent effects
- spasmolytic effects
- secretolytic effects
- antitussive effects
- other effects on the gastro-intestinal tract, the respiratory tract or the immune system
- treatment of diarrhea, bacterial or viral infections of the gastro-intestinal tract

or respiratory tract, broncho-pneumonia, common cold, cough

(b)

- ingredients, constituent, components of plants and the detection

or extraction of them

- toxic activity or side effects

10.2. Exclusion criteria:

Exclusion of all references which

- do not provide an abstract
- are only presented on conferences and not in peer-reviewed journals
- investigate a mixture of different plant species as a combined preparation
- investigating antioxidant effects
- dealing with other plant species or subspecies than the focused
- state no clear result in the abstract

or dealing with

- other animal classes than mammalians and birds
- other medical branches of study, other diseases or apparatuses than mentioned in the inclusion criteria (e.g. dermatology, cardiology, oncology, nephrology, diabetes…)
- other pathogens than the main pathogens connected with gastrointestinal or respiratory diseases in calves and piglets
- plants used as food
- plant genetics
- cultivation or breeding of plants
- plant pathology, plant protection systems or pesticides
- ecology
- geology
- ethology
- sociology
- food technology or food-packaging
- the use of the plant as a repellent or insecticide

11. Data processing

11.1. Classification of the references regarding

(a) different therapeutic needs based on pathophysiology and etiology of the focus diseases and

(b) the investigated effects of plant preparations.

Classification of therapeutic needs: antibacterial, antiviral, antiprotozoal, spasmolytic, anti-inflammatory, anti-adhesive, antidiarrheic, immunostimulant, analgesic, expectorant or antitussive effects or an enhancement of antibiotics.

11.2. Classification of the final remaining references in predefined categories referring with the type of study:

- *in vitro* studies = all studies conducted on cellular level and ex vivo studies
- *in vivo* studies = all studies using an animal model, which normally is not suffering from the disease / health problem
- clinical trials = all studies using an animal species (including human), which may suffer from the disease / health problem
- reviews = assessment and comparison of available literature on one topic
- pharmacognostic studies = all studies aiming the biochemical composition or the analysis of their components, toxicity and side-effects of plants

12. Assessment of the plant species with a scoring system

An investigated effect is defined as ‘proven’, if the reference states that the investigated plant species shows significantly the effect in *in vitro* or *in vivo* models or in clinical studies. For each significantly proven hypothesized effect, the plant gets 1 point (marked as “+”)

The phrase ‘uncertain effect’ is used, if the plant species is only active against single pathogens and not against all investigated ones, if the hypothesized effect is shown evidently only in some replicates or if only one aspect of the hypothesized effect is shown evidently. For each uncertain effect, the plant gets no points (marked as “?”).

For each refutation of hypothesized effect or if no effect significantly can be shown, the effect is defined as disproved. For each ‘disproof of effect’ one point is subtracted (marked as “o”).

The summation of all points for each plant within each type of study (clinical/*in vivo*/*in vitro*) and each indication (QA/QR/QL) follows the formula:

Score = 3 x (number of proven effects in clinical studies – number disproof of effects in clinical studies)+ 2 x (number of proven effects in *in vivo* studies - number of disproof of effects in *in vivo* studies) + 1 x (number of proven effects in *in vitro* studies - number of disproof of effects of *in vitro* studies)

According to this scoring system a list of most promising plant species for the prophylaxis and treatment of gastrointestinal and respiratory diseases of calves and piglets is established.
